# Estimating the basic reproduction number for COVID-19 in Western Europe

**DOI:** 10.1371/journal.pone.0248731

**Published:** 2021-03-17

**Authors:** Isabella Locatelli, Bastien Trächsel, Valentin Rousson

**Affiliations:** Center for Primary Care and Public Health (Unisanté), University of Lausanne, Lausanne, Switzerland; Centers for Disease Control and Prevention, UNITED STATES

## Abstract

**Objective:**

To estimate the basic reproduction number (*R*_0_) for COVID-19 in Western Europe.

**Methods:**

Data (official statistics) on the cumulative incidence of COVID-19 at the start of the outbreak (before any confinement rules were declared) were retrieved in the 15 largest countries in Western Europe, allowing us to estimate the exponential growth rate of the disease. The rate was then combined with estimates of the distribution of the generation interval as reconstructed from the literature.

**Results:**

Despite the possible unreliability of some official statistics about COVID-19, the spread of the disease appears to be remarkably similar in most European countries, allowing us to estimate an average *R*_0_ in Western Europe of 2.2 (95% CI: 1.9–2.6).

**Conclusions:**

The value of *R*_0_ for COVID-19 in Western Europe appears to be significantly lower than that in China. The proportion of immune persons in the European population required to stop the outbreak could thus be closer to 50% than to 70%.

## Introduction

The COVID-19 pandemic is an ongoing pandemic of coronavirus disease caused by acute respiratory syndrome coronavirus 2 (SARS-CoV-2). It was first identified in December 2019 in Wuhan, China. Spreading rapidly around the world [1], it was declared a public health emergency of international concern in January 2020 and a pandemic in March 2020 when all inhabited continents and more than 200 countries were affected. By early 2021, more than 90 million cases were confirmed worldwide and more than 2 million deaths were attributed to COVID-19.

The basic reproduction number (*R*_0_) is a well-known epidemiological concept to measure the spread of an infectious disease [2–5]. It is defined as the average number of secondary cases that one primary case will generate in a given population, where nobody is either immune or vaccinated. It is thus defined at the start of an outbreak, in particular, before any public health measure is undertaken. A value of *R*_0_ above 1 implies an exponential growth in the number of cases of the disease in the population, while a value of *R*_0_ below 1 indicates that the outbreak will stop. As a consequence, the epidemic will also stop once the proportion *P* of immune (or vaccinated) persons in the population reaches value 1−1/*R*_0_, which guarantees an “effective reproduction number” of *R* = *R*_0_(1−*P*) that is smaller than one. It is thus of interest to estimate the value of *R*_0_ for an emerging disease, such as the current COVID-19 outbreak.

Most reported *R*_0_ for COVID-19 were estimated using Chinese data. A comprehensive meta-analysis including 29 studies about China reported an estimated value of *R*_0_ = 3.32 (95% CI: 2.81−3.82) [6], indicating that the proportion of immune persons needed to stop the outbreak would be close to 70%. However, as explained e.g. by Delamater et al. [7], the value of *R*_0_ is essentially the combination of three factors: the (average) number of daily contacts that one contagious person has, the probability of transmission of the disease during such a contact, and the (average) number of days that an infected person is contagious. While the latter factor mainly depends on the biological characteristics of the disease, the first two factors strongly depend on the social habits of a given population. Since these habits may vary considerably in countries with different cultures, an estimated value of *R*_0_ in China is not necessarily valid in Europe.

To date, published studies reporting an *R*_0_ for COVID-19 in Europe are rare. The recent meta-analysis by Billah et al [8] included only three studies with European data, the first providing an estimate of *R*_0_ in France, Germany, Italy and Spain [9], the second in Spain, where an effective reproduction number was also estimated after a lockdown was declared [10], the third focusing on a single Italian region [11]. Another study by Hilton and Keeling [12] used a sophisticated methodology to combine epidemiological data from China with country-specific “synthetic contact matrices” measuring the density of human contact networks, to estimate an *R*_0_ in 152 countries, including most European ones. A typical difficulty in studies estimating an *R*_0_ is the possible unreliability of the epidemiological data used [13]. The goal of the present study is to estimate a value of *R*_0_ for the COVID-19 outbreak in Western Europe that is as reliable as possible by combining data from several countries. We thus apply the principle of “borrowing strength” [14], trying to consolidate the partly unreliable data collected in some countries by pooling them with better data collected in other (albeit similar) countries. To do this, we robustly estimate the exponential growth rate of the disease in 15 Western European countries based on the number of reported daily incidences (new cases) and then average them to obtain an overall estimate of the exponential growth rate for Western Europe. To obtain an estimated value of *R*_0_, the latter estimation is combined with an estimation of the parameters of the generation interval distribution, i.e., the time needed for an infected person (primary case) to infect another person (secondary case), as reconstructed from the literature.

## Data

**Fig 1:**
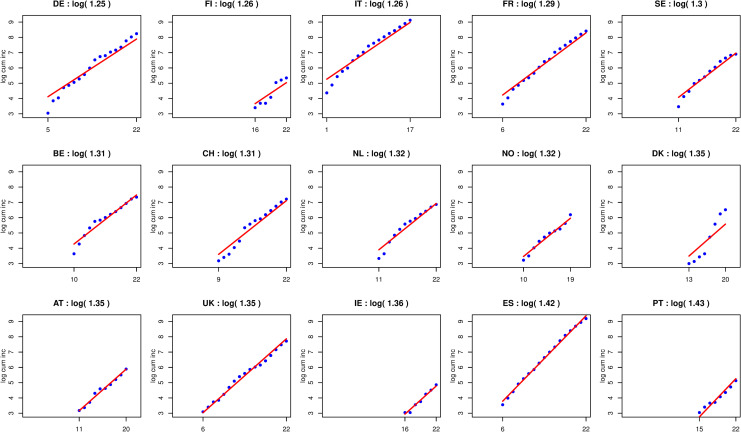
Cumulative incidence of COVID-19 over a selected period in 15 countries in Western Europe (log scale). For each country, a line has been added to the plot, whose intercept represents the center of gravity of the data, and whose slope represents the estimated exponential growth rate of the disease at the start of the outbreak. The selected period was different for each country, ranging from February 23 (day 1) to March 15 (day 22).

We downloaded incidence data for COVID-19 from the EU Open Data Portal on September 30, 2020 (https://data.europa.eu/euodp/en/data/dataset/covid-19-coronavirus-data). We used the daily numbers of new COVID-19 cases from the start of the outbreak until (and including) March 15, 2020, as reported in all the 15 countries in Western Europe with at least one million inhabitants (https://fr.m.wikipedia.org/wiki/Europe_de_l%27Ouest): Austria (AT), Belgium (BE), Switzerland (CH), Germany (DE), Denmark (DK), Spain (ES), Finland (FI), France (FR), Ireland (IE), Italy (IT), the Netherlands (NL), Norway (NO), Portugal (PT), Sweden (SE) and the United Kingdom (UK). Note that it was important to consider a period at the very beginning of the COVID outbreak, at a time when social habits were not affected by the pandemic and its consequences. We included data until March 15 to ensure that all selected countries had data for at least 7 days (see below). From there, we calculated the cumulative incidence of COVID-19 for a given day in a given country by summing all the new cases observed up to and including that day in that country. The obtained daily cumulative incidence numbers are plotted on Fig 1 for the 15 countries on a (natural) logarithmic scale, where they are supposed to be aligned at the start of an outbreak that is growing exponentially, which was largely the case here, validating the choice of our selected period.

Since incidences based on too few cases are unreliable, the standard error (and thus statistical imprecision) of the logarithm of an incidence being inversely proportional to the square root of the number of cases [15, p. 238], only daily cumulative incidence values greater than or equal to 20 were retained in our analysis, as done in Musa et al [16] to estimate an exponential growth rate in Africa. The first country to reach this milestone was Italy on February 23, 2020, which is referred to as day 1 in the graphics. March 15, 2020 is thus day 22. The last countries to reach this milestone were Finland and Ireland (on day 16, i.e., on March 9, 2020). Of note, no data were available on March 10 in Ireland and on March 11 in Finland (we thus assumed that no new cases were reported). We also excluded from our analysis those data collected during a time period after a national confinement had been declared. Before March 15, this was the case in Austria (March 13), Denmark (March 13), Norway (March 12) and Italy (March 10). Thus, the number of days included in our analysis varied from country to country, ranging from 7 days (for Finland and Ireland) to 17 days (for Spain, France, Italy and the UK) or even 18 days (for Germany).

## Methods

The exponential growth rate *ρ* of a disease in a population is defined as the daily increase in the cumulative incidence, calculated on the natural logarithmic scale, at the start of an outbreak. Thus *ρ* = *log*(*N*_*j*+1_)−log(*N*_*j*_) = log(*N*_*j*+1_/*N*_*j*_), where *N*_*j*_ is the cumulative incidence observed on day *j* in the examined population. Therefore, exp(*ρ*) = *N*_*j*+1_/*N*_*j*_ represents the daily increase (expressed as a percentage) of the cumulative incidence. For example, a value of *ρ* = 0.25 indicates that one can multiply the cumulative incidence observed on a given day by exp(*ρ*) = 1.28 to obtain the cumulative incidence for the next day (where the daily increase is 28%). To get an estimate $\hat{\rho }$ of *ρ*, one must thus average (or smooth) the ratios *N*_*j*+1_/*N*_*j*_ over a selected period. One possible method for estimating *ρ* is to consider the slope of a Poisson regression of log(*N*_*j*_) on day *j* (including all days *j* in the selected period), as done e.g. in Yuan et al [9]. However, this method might not be robust to the unreliability of some data; for example, a “weekend effect”, where a few cases not reported during a weekend might be reported on the following Monday, might skew the data and negatively affect the model. This is why we estimated *ρ* in each country as the (natural) logarithm of the median of the empirical ratios *N*_*j*+1_/*N*_*j*_ observed over the selected period. This method is robust to unreliability of some data provided that half of the successive cumulative incidences reported in a given country are reliable. To obtain an overall estimate $\hat{\rho }$ of *ρ* in Western Europe, the estimations of *ρ* obtained in each country were averaged across all 15 countries. In a same spirit as in a meta-analysis, we used a weighted average, where the weight assigned to a country was proportional to the number of days included in the analysis for that country.

Let now $g(t;\mu ,\sigma_{G}^{2})$ be the density function of the *generation interval G*, where a generation interval is defined as the time needed for an infected person to infect another person with COVID-19 (expressed in days), while *μ* and $\sigma_{G}^{2}$ indicate the mean and variance of *G*, respectively. As explained in detail by Wallinga and Lipsitch [17], knowing the distribution of *G* allows relating the exponential growth parameter *ρ* with *R*_0_ via the following formula:

$$R_{0}=\frac{1}{\int_{0}^{\infty }\exp\left(-\rho t\right)g\left(t;\mu ,\sigma_{G}^{2}\right)dt}.$$ (1)

The distribution of *G* and its parameters are usually estimated empirically by considering confirmed infector-infected pairs [1, 18–22]. However, since a diseased subject usually comes under observation only from the onset of symptoms, one in general only disposes of the time difference between the dates when the infector and infected show symptoms, referred to as the *serial interval S*. This is why in most studies the distribution of *S* is taken as a proxy of the distribution of *G* in (1) [18, 22]. However, as acknowledged by Ganyani et al. [19], while the distributions of *G* and *S* should have the same mean *μ*, the variance $\sigma_{S}^{2}$ of *S* is in general larger than $\sigma_{G}^{2}$. In addition, while *S* may happen to be negative (when the infected person develops symptoms before the infector) and may exhibit a symmetric (e.g. close to normal) distribution [18], *G* is always positive by definition and is typically characterized by a distribution that is skewed to the right, such as a Weibull or a gamma distribution.

The relationship between $\sigma_{G}^{2}$ and *R*_0_, which is not immediately evident from (1), can be highlighted by considering a distribution with an explicit solution of the integral to the denominator of (1), which is the moment generating function of the random variable *G*. A convenient example is the gamma distribution. In that case, expression (1) reduces to:

$$R_{0}=exp\left[\frac{\mu^{2}}{\sigma_{G}^{2}}log\left(1+\frac{\sigma_{G}^{2}}{\mu }\rho \right)\right].$$ (2)

One can easily verify that expression (2) is a decreasing function of $\sigma_{G}^{2}$, converging to the well-known approximation *R*_0_≈*exp*(*μρ*) which is sometimes used in the literature to estimate *R*_0_ [23]. Thus, underestimating the variability of *G* in (1) or (2) leads to overestimating *R*_0_, the bias being at its maximum when the variance $\sigma_{G}^{2}$ is set to 0, while overestimating the variability of *G* (as we do if we consider the distribution of *S* as a proxy of the distribution of *G*) leads to underestimating *R*_0_.

It is thus important to get a correct estimate of $\sigma_{G}^{2}$. To achieve this from an estimate of $\sigma_{S}^{2}$, we consider that *G* and *S* are related as follows:

$$S=G+\left(I_{2}-I_{1}\right).$$ (3)

In (3), *I*_1_ and *I*_2_ refer to the *incubation period*, i.e., the time between the infection and the onset of symptoms in a diseased subject for the infector and for the infected, respectively. If we denote by $\sigma_{I}^{2}$ the variance of an incubation period, we thus have from (3):

$$\sigma_{G}^{2}=\sigma_{S}^{2}-2\sigma_{I}^{2}.$$ (4)

An estimate ${\hat{\sigma }}_{G}^{2}$ of $\sigma_{G}^{2}$ was thus obtained as ${\hat{\sigma }}_{G}^{2}={\hat{\sigma }}_{S}^{2}-2{\hat{\sigma }}_{I}^{2}$, combining according to (4) an estimate ${\hat{\sigma }}_{S}^{2}$ of the serial interval variance found in a study of infector-infected pairs, with an estimate ${\hat{\sigma }}_{I}^{2}$ of the incubation period variance obtained in a study of diseased subjects for whom the dates of infection (and the symptoms onsets dates) are available. An estimate $\hat{\mu }$ of the mean of *S* (corresponding to the mean of *G*) was also taken from the literature on infector-infected pairs. Our estimation ${\hat{R}}_{0}$ of the basic reproductive number was then obtained as:

$${\hat{R}}_{0}=\frac{1}{\int_{0}^{\infty }\exp\left(-\hat{\rho }t\right)g\left(t;\hat{\mu },{\hat{\sigma }}_{G}^{2}\right)dt}.$$ (5)

In (5), we assumed either a Weibull or a gamma distribution for *G*. With a gamma distribution, we could use the explicit form (2), whereas a numerical integration was used in the case of the Weibull distribution, for which the moment generating function has no explicit form.

Confidence intervals for *R*_0_ were obtained based on 10’000 simulations from the sampling distributions of the estimates [24, 25], as detailed in the S1 Appendix. Our code was written using the R statistical software [26] and is available upon request.

## Results

Fig 1 shows the cumulative incidences of COVID-19 (and thus all the data used in this analysis) on the logarithmic scale over the selected period for the 15 countries. As already mentioned, the data were remarkably aligned (as they should be at the start of an exponentially growing outbreak), with slopes that were similar for most countries. Our estimation of the exponential growth rates *ρ* ranged from log(1.25) for Germany to log(1.42) and log(1.43) for Spain and Portugal, the weighted average over the 15 countries being $\hat{\rho }$ = log(1.32) (95% CI: log(1.29)-log(1.35)).

Reliable estimates of the mean and variance of the serial interval *S* can be found in Du et al. [18] based on a large number of infector-infected pairs (n = 468). Using a normality assumption for *S*, they estimated a mean $\hat{\mu }=3.96\;(95\%\;CI:\;3.53-4.39)$ and a variance of ${\hat{\sigma }}_{S}^{2}=4.75^{2}\;(95\%\;CI:\;4.46^{2}-5.07^{2})$. Concerning the variance of the incubation period *I*, we used the estimate ${\hat{\sigma }}_{I}^{2}=2.3^{2}\;(95\%\;CI:\;1.7^{2}-3.7^{2})$ obtained by Backer et al. [27] based on n = 88 diseased subjects for whom the dates of infection could be retrieved, assuming a Weibull distribution (which yielded a better fit than that of a gamma distribution). Putting these results together as explained in the Methods section and by assuming a Weibull distribution for *G*, we obtained an estimate of its variance of ${\hat{\sigma }}_{G}^{2}=3.46^{2}\;(95\%\;CI:\;2.41^{2}-4.95^{2})$, and finally of the basic reproductive number for COVID-19 in Western Europe, which is given by ${\hat{R}}_{0}=2.21\;(95\%\;CI:\;1.86-2.63).$

Table 1 provides some alternative estimates of *R*_0_ by assuming gamma instead of Weibull distributions for *I* and/or *G*. While considering a gamma (instead of a Weibull) distribution for *G* had almost no influence, our estimate of *R*_0_ increased slightly (from 2.2 to 2.3) when we considered a gamma (instead of a Weibull) distribution for *I*. This was due to a slight increase of estimate ${\hat{\sigma }}_{I}=2.6\;(95\%\;CI:\;1.8-4.2)$ found in Backer et al. [27] in the case of a gamma distribution, leading in turn to a decrease of estimate ${\hat{\sigma }}_{G}=3.01\;(95\%\;CI:\;1.57-5.71)$.

**Table 1:** 

*I*	*G*	${\hat{R}}_{0}$
Weibull	Weibull	2.21 (95% CI : 1.86–2.63)
Weibull	Gamma	2.22 (95% CI: 1.84–2.64)
Gamma	Weibull	2.32 (95% CI: 1.77–2.89)
Gamma	Gamma	2.34 (95% CI: 1.73–2.91)

Summary of the estimates ${\hat{R}}_{0}$ of the basic reproductive number using various assumptions for the distributions of the incubation period *I* and generation interval *G*.

## Discussion

The value of *R*_0_ for an infectious disease, such as COVID-19, depends not only on the biological characteristics of the disease but also on the social habits of the population and might thus be different from country to country. In this paper, we provide an estimation of *R*_0_ for COVID-19 in Western Europe. To achieve this, we have combined the cumulative incidences of COVID-19 reported at the start of the outbreak in the 15 largest countries in Western Europe, with an estimation of the parameters of the generation interval distribution reconstructed from the literature.

In contrast with other authors [18, 22] who used the parameters of the serial interval distribution as a proxy for those of the generation interval distribution, we have reconstructed the latter from estimates of the former, also integrating an estimate of the variance of the incubation period in a similar spirit as Ganyani et al. [19]. As these authors do, and as is also common in applications in chemistry or physics [28], we have combined estimates from different (and independent) data sources, looking for the best possible (most reliable) estimate for each biological parameter. In particular, the reliable estimates of these quantities, i.e., estimates based on large data sets, that we were able to find in the literature used Chinese, and not European data, which is a limitation of our study. However, the incubation period is mainly a biological characteristic, which should be similar in most countries of the world. The generation interval (and thus the serial interval) also depends (among other characteristics) on the viral load, another biological characteristic, so we hope that the estimates obtained in China also largely apply in Europe. Nevertheless, researchers can still update our calculations using estimates of the mean and variance of the serial interval based on European data (once available). On the other hand, we found that the shape of the distribution (e.g., Weibull or gamma) of the generation interval had only a minimal impact on our final estimates.

As mentioned in the Introduction, most studies about *R*_0_ for COVID-19 were done for (various regions of) China [22], for which a meta-analysis reported an estimate of 3.3 for *R*_0_ [6]. Our estimate of 2.2 is thus significantly lower than that in China. This might be due to a higher number of daily contacts for a citizen in China than for a citizen in Europe because of the dense populations in Chinese cities. This might also be due to methodological issues, since, as mentioned in our Methods section, ignoring the variance of the generational interval leads to an overestimation of *R*_0_. In contrast, in one of the rare studies performed in Europe, *R*_0_ was estimated to be approximately 3.3, 6.3, 6.1 and 5.1 in Italy, France, Germany and Spain, respectively [9]. However, the authors used the incubation period as a proxy for the generational interval. This is quite questionable, particularly since the incubation period for COVID-19 typically has a larger mean than the generation (as well as the serial) interval (e.g., 6 days instead of 4 days).

In an impressive study combining epidemiological data from China with synthetic contact matrices to capture country-specific contact behavior, Hilton and Keeling [12] estimated an *R*_0_ in 152 countries, including 14 out of the 15 European countries considered here (Norway was not included). An average of their estimates of *R*_0_ over these 14 countries would be 2.1 if we consider their simpler model, and 3.0 if we consider their more complex model, their estimates being highly dependent of the complexity of the mathematical model used, as recognized by the authors. In contrast, our estimate was obtained in a relatively simple and totally transparent manner.

We are not claiming that the value of *R*_0_ is the same in each European country. However, despite some legitimate doubt about the quality and reliability of some official statistics on the incidence of COVID-19 in some countries and despite the different testing strategies that were applied in different European countries, the spread of the disease appears to be remarkably similar in almost all countries, as shown in Fig 1. In such a homogeneous context, calculating an “average *R*_0_” to summarize the spread of the disease in Western Europe appears relevant to us, justifying the application of the principle of “borrowing strength” mentioned in the Introduction.

In summary, the value of *R*_0_ for COVID-19 in Western Europe might be slightly lower than the values that are sometimes reported in other countries (e.g., on Wikipedia, where the reported values of *R*_0_ are up to 6). A practical consequence of this is that the proportion of immune persons in the European population required to stop the outbreak is estimated to be 1-1/2.2 = 55%, which is closer to 50% than to 70%. This might be a useful message at a time when the COVID-19 outbreak is not yet over.

## Supporting information

S1 Appendix (DOCX)
