# Supplementary material for: Estimating the basic reproduction number for COVID-19 in Western Europe
Source: PLoS One. 2021 Mar 17;16(3):e0248731. doi: 10.1371/journal.pone.0248731 (PMC7968714; doi:10.1371/journal.pone.0248731)
Supplement: S1 Appendix — (DOCX) [file pone.0248731.s001.docx]

# S1 APPENDIX.

Our estimate $\hat{R}_{0}$ of $R_{0}$ given by equation (5) depends on three estimates: an estimate $\hat{\rho}$of the exponential growth rate, and estimates $\hat{\mu}$ and $\hat{\sigma}_{G}^{2}$ of the mean and variance of the generation interval. As explained in the Methods section, the latter estimate was obtained as $\hat{\sigma}_{G}^{2}=\hat{\sigma}_{S}^{2}-2\hat{\sigma}_{I}^{2}$ $,$ and thus depended itself on two other estimates: an estimate of the serial interval variance $\hat{\sigma}_{S}^{2}$and an estimate of the incubation period variance $\hat{\sigma}_{I}^{2}.$ While $\hat{\rho}$ was calculated in our own study, together with its variance $V(\hat{\rho})$, the other three estimates $\hat{\mu}$, $\hat{\sigma}_{S}^{2}$and $\hat{\sigma}_{I}^{2}$ were obtained from the literature, where 95% confidence intervals ($\hat{\mu}_{L},\hat{\mu}_{U}$), $(\hat{\sigma}_{SL}^{2},\hat{\sigma}_{SU}^{2})$ and $(\hat{\sigma}_{IL}^{2},\hat{\sigma}_{IU}^{2})$ were also available. Assuming normality of $\hat{\mu}$, ${log\hat{\sigma}}_{S}^{2}$ and ${log\hat{\sigma}}_{I}^{2}$ (thanks to the central limit theorem and the reasonably large sample sizes), one can easily deduce their variances $V\left( \hat{\mu} \right)$, $V\left( {log\hat{\sigma}}_{S}^{2} \right)$ and $V\left( {log\hat{\sigma}}_{I}^{2} \right)$as follows:

$$V\left( \hat{\mu} \right)=\left[ \frac{\hat{\mu}_{U}-\hat{\mu}_{L}}{2\cdot1.96} \right]^{2},$$

$$V\left( {log\hat{\sigma}}_{S}^{2} \right)=\left[ \frac{log\hat{\sigma}_{SU}^{2}-log\hat{\sigma}_{SL}^{2}}{2\cdot1.96} \right]^{2},$$

$$V\left( {log\hat{\sigma}}_{I}^{2} \right)=\left[ \frac{log\hat{\sigma}_{IU}-log\hat{\sigma}_{IL}}{2\cdot1.96} \right]^{2} .$$

An approximation of the variance $V\left( {log\hat{\sigma}}_{G}^{2} \right)$ of ${log\hat{\sigma}}_{G}^{2}$ was then calculated as:

$${V(log\hat{\sigma}}_{G}^{2})\cong\frac{V\left( {log\hat{\sigma}}_{S}^{2} \right)\left( \hat{\sigma}_{S}^{2} \right)^{2}+4V\left( {log\hat{\sigma}}_{I}^{2} \right)\left( \hat{\sigma}_{I}^{2} \right)^{2}}{\left( \hat{\sigma}_{S}^{2}-2\hat{\sigma}_{I}^{2} \right)^{2}} . (A1)$$

Expression (A1) was obtained applying the following Taylor approximations:

1. ${V(log\hat{\sigma}}_{G}^{2})\cong V\left[ log\left( \hat{\sigma}_{S}^{2}-2\hat{\sigma}_{I}^{2} \right) \right]=\left[ V\left( \hat{\sigma}_{S}^{2} \right)+4V\left( \hat{\sigma}_{I}^{2} \right) \right]/\left( \hat{\sigma}_{S}^{2}-2\hat{\sigma}_{I}^{2} \right)^{2}$
2. $V\left( \hat{\sigma}_{S}^{2} \right)=V\left[ \exp({log\hat{\sigma}}_{S}^{2}) \right]\cong V\left( {log\hat{\sigma}}_{S}^{2} \right)\left[ \exp\left( {log\hat{\sigma}}_{S}^{2} \right) \right]^{2}=V\left( {log\hat{\sigma}}_{S}^{2} \right)\left( \hat{\sigma}_{S}^{2} \right)^{2}$
3. $V\left( \hat{\sigma}_{I}^{2} \right)=V\left[ \exp({log\hat{\sigma}}_{I}^{2}) \right]\cong V\left( {log\hat{\sigma}}_{I}^{2} \right)\left[ \exp\left( {log\hat{\sigma}}_{I}^{2} \right) \right]^{2}=\left( {log\hat{\sigma}}_{I}^{2} \right)\left( \hat{\sigma}_{I}^{2} \right)^{2}$

We then simulated 10’000 replications of the three estimates $\hat{\rho}$, $\hat{\mu}$ and ${log\hat{\sigma}}_{G}^{2}$ involved in equation (5) according to their sampling distributions: $N\left( \hat{\rho} ,V\left( \hat{\rho} \right) \right)$, $N\left( \hat{\mu},V\left( \hat{\mu} \right) \right)$ and $N\left( {log\hat{\sigma}}_{G}^{2},V\left( {log\hat{\sigma}}_{G}^{2} \right) \right)$. Note that such distributions can be interpreted either in a Bayesian spirit as posterior distributions of the unknown parameters, or in a frequentist maximum likelihood perspective as sampling distributions of the parameter estimates [24]. Combining the above sampled estimates according to (5) yields the sampling distribution of $\hat{R}_{0}$. A 95% confidence interval for $R_{0}$ was finally formed by the 2.5% and 97.5% quantiles of the sampling distribution of $\hat{R}_{0}$ [25].
